# Use of neoadjuvant electrochemotherapy to treat a large metastatic lesion of the cheek in a patient with melanoma

**DOI:** 10.1186/1479-5876-10-131

**Published:** 2012-06-22

**Authors:** Nicola Mozzillo, Corrado Caracò, Stefano Mori, Gianluca Di Monta, Gerardo Botti, Paolo A Ascierto, Corradina Caracò, Luigi Aloj

**Affiliations:** 1 Department of Surgery “Melanoma - Soft Tissues - Head & Neck - Skin Cancers”, National Cancer Institute, Via Mariano Semmola, Naples, Italy; 2 Department of Pathology, National Cancer Institute, Via Mariano Semmola, Naples, Italy; 3 Medical Oncology and Innovative Therapies Unit, National Cancer Institute, Via Mariano Semmola, Naples, Italy; 4 Department of Nuclear Medicine, National Cancer Institute, Via Mariano Semmola, Naples, Italy

**Keywords:** Electrochemotherapy, Melanoma, Adjuvant treatment

## Abstract

**Background:**

Approximately 200,000 new cases of melanoma are diagnosed worldwide each year. Skin metastases are a frequent event, occurring in 18.2% of cases. This can be distressing for the patient, as the number and size of cutaneous lesions increases, often worsened by ulceration, bleeding and pain. Electrochemotherapy (ECT) is a local modality for the treatment of cutaneous or subcutaneous tumors that allows delivery of low- and non-permeant drugs into cells. ECT has been used in palliative management of metastatic melanoma to improve patients’ quality of life. This is, to our knowledge, the first application of ECT as neoadjuvant treatment of metastatic subcutaneous melanoma.

**Methods and results:**

A 44-year-old Caucasian woman underwent extensive surgical resection of a melanoma, with a Breslow thickness of 1.5 mm, located on the right side of her scalp. No further treatment was given and the woman remained well until she came to our attention with a large nodule in her right cheek. Whole-body fluorodeoxyglucose positron emission tomography/computed tomography (FDG PET/CT) was performed for staging and treatment monitoring. Baseline FDG PET/CT showed the lesion in the cheek to have a maximal standardized uptake value (SUVmax) of 19.5 with no evidence of further disease spread. Fine needle aspiration cytology confirmed the presence of metastatic melanoma. The patient underwent two sessions of ECT with intravenous injections of bleomycin using a Cliniporator^TM^ as neoadjuvant treatment permitting conservative surgery three months later.

Follow-up PET/CT three months after the first ECT treatment showed a marked decrease in SUVmax to 5. Further monitoring was performed through monthly PET/CT studies. Multiple cytology examinations showed necrotic tissue. Conservative surgery was carried out three months after the second ECT. Reconstruction was easily achieved through a rotation flap. Pathological examination of the specimen showed necrotic tissue without residual melanoma. One year after the last ECT treatment, the patient was disease-free as determined by contrast-enhanced CT and PET/-CT scans with a good functional and aesthetic result.

**Conclusions:**

ECT represents a safe and effective therapeutic approach that is associated with clear benefits in terms of quality of life (minimal discomfort, mild post-treatment pain and short duration of hospital stay) and may, in the neoadjuvant setting as reported here, offer the option of more conservative surgery and an improved cosmetic effect with complete local tumor control.

## Background

Approximately 200,000 new cases of melanoma are diagnosed worldwide each year [1,2]. Skin metastases are a frequent event, occurring in 18.2% of cases [3]. This can be distressing for the patient, as the number and size of cutaneous lesions increases, often worsened by ulceration, bleeding and pain. Electrochemotherapy (ECT) is a local modality for the treatment of cutaneous or subcutaneous metastases that allows delivery of low- and non-permeant drugs into cells [4]. ECT has been used in palliative management of metastatic melanoma to improve patients’ quality of life [5-9]. This is, to our knowledge, the first application of ECT as neoadjuvant treatment of metastatic subcutaneous melanoma.

## Methods and results

**Figure 1:**
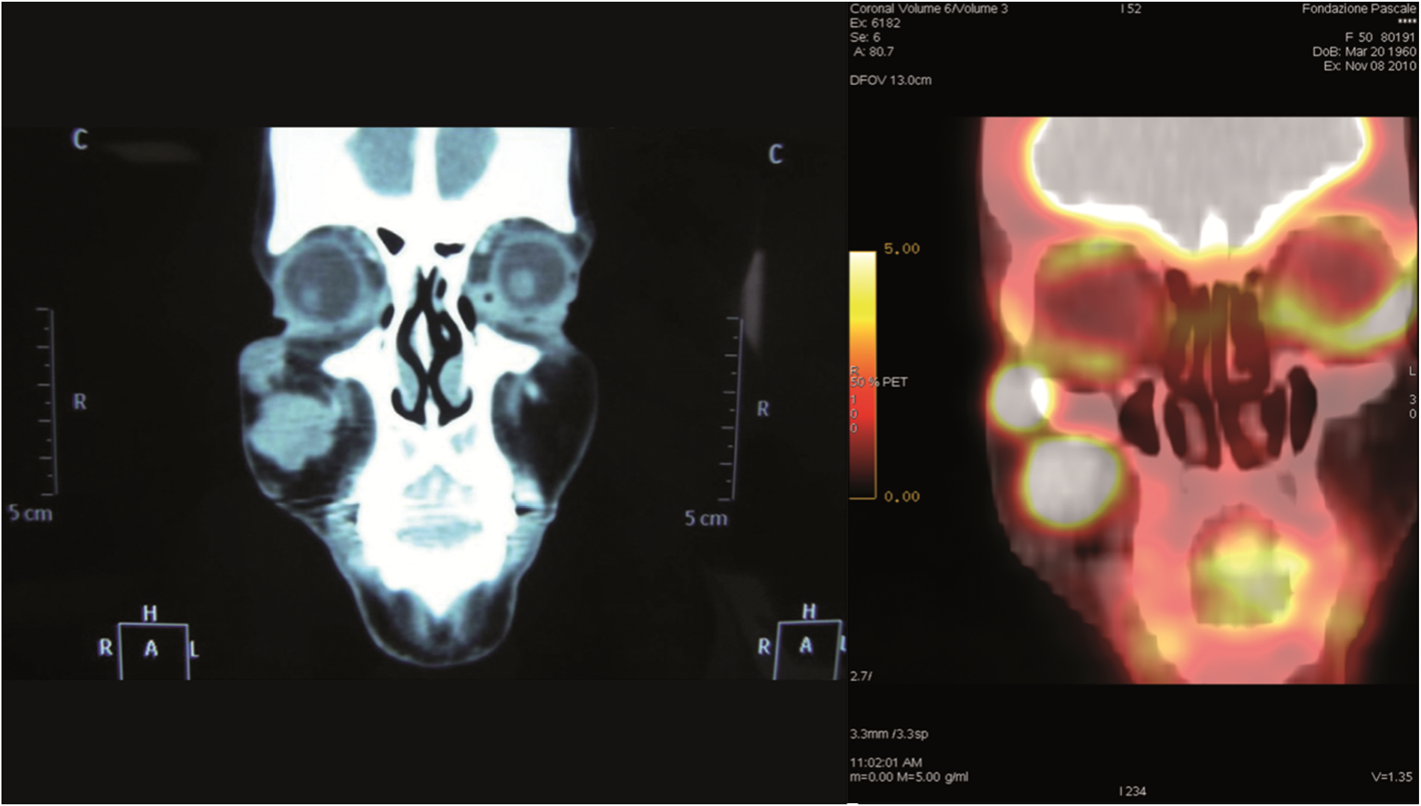
Pre-treatment CT scan with evidence of sandglass-shaped mass of the right cheek and FDG-PET scan with a SUVmax of 19.5.

A 44-year-old Caucasian woman underwent extensive surgical resection of a melanoma, with a Breslow thickness of 1.5 mm, located on the right side of her scalp. No further treatment was given and the woman remained well until she presented to our attention when a large nodule appeared in her right cheek. Physical examination revealed a 3.5 cm maximum diameter lesion partly adhered to the adjacent overlying skin but with no apparent infiltration of the inner oral mucosa, as confirmed by computed tomography (CT) scan. The whole-body CT scan revealed a 3.5 cm maximum diameter sandglass-shaped large mass, engaging the right cheek, with the upper extremity close to the floor of the orbit. No other metastatic localization was detected. Whole-body fluorodeoxyglucose positron emission tomography/CT (FDG PET/CT) was performed for staging and treatment monitoring under standardized conditions that included fasting for at least 6 h, administration of 3.7 MBq/kg of FDG and imaging one hour after injection. The baseline FDG PET/CT study showed the lesion in the cheek to have a maximal standardized uptake value (SUVmax) of 19.5 with no evidence of further disease spread (Figure 1). Fine needle aspiration cytology confirmed the presence of metastatic melanoma.

Standard radical surgery would have required a large resection of the whole cheek, with wide safety margins of the mass, including the skin and oral mucosa surface, branches of the mandibolar facial nerve and part of the orbit floor. Instead, the patient was spared this and underwent two sessions of ECT using a Cliniporator^TM^ (IGEA S.p.A, Carpi, Italy) as elective treatment. The patient signed a detailed informed consent for the different therapeutic options. ECT treatment was performed after the approval of an appropriate ethics committee (IEC of National Cancer Institute of Naples, reference number 273/10) in compliance with Helsinki Declaration, following internationally recognized guidelines. The first neoadjuvant ECT treatment was performed with the patient receiving intravenous (IV) bleomycin 15000 IU/m [2] under general anesthesia. The second ECT session was performed six weeks later with the same dose of drug. In both treatments, the procedure was started 8 min after IV drug injection with linear configuration needle electrodes (type II electrodes, length 20 mm, 4 mm distance between rows, IGEA S.p.A, Carpi, Italy) being used. Twelve and 8 electric pulses were delivered in the first and second ECT sessions, respectively. In both, treatment was performed without dual application on the same area so as to reduce the risk of necrosis. No post-treatment complications and no peripheral nerves injuries were observed. The first follow-up FDG PET/CT scan was performed three months after the second ECT. This showed marked improvement compared with the baseline scan and the SUVmax of the lesion had decreased to 5. Further monitoring was performed through monthly PET/CT. Multiple cytology examinations showed necrotic tissue.

**Figure 2:**
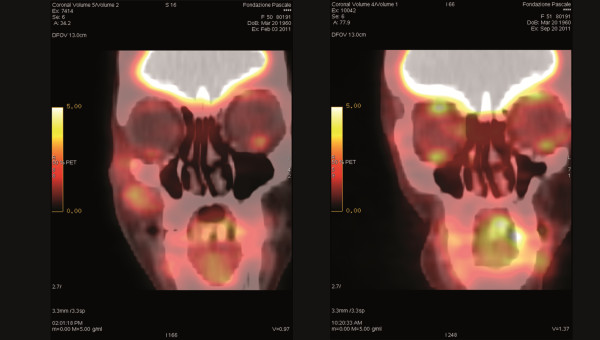
(Left) Post electrochemotherapy and (right) post surgical treatment FDG-PET scan with SUVmax respectively decreased to 5 and to 1,3.

**Figure 3:**
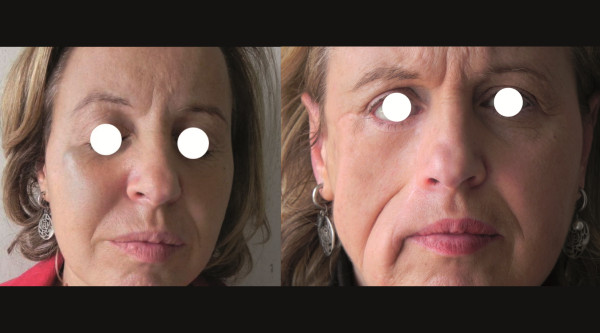
Pre–treatment clinical image and post-operative image with a good functional and aesthetic result.

Conservative surgery was carried out three months after the second ECT, and the mass excised, including a narrow rim of healthy tissue margin and a small triangle of adherent skin, sparing the inner layer, the oral mucosa and the floor of the orbit. Reconstruction was easily achieved through a rotation flap. Pathological examination of the specimen, which included a solid lesion with a maximum diameter of 1.3 cm, showed necrotic tissue without residual melanoma. Six months after surgery, the SUVmax in the region of the lesion had further decreased to 1.3 (Figure 2). One year after the last ECT, the patient was disease-free as determined by CT and PET/CT scans with a good functional and aesthetic result (Figure 3).

## Discussion

Application of short and high-intensity electric pulses (reversible electroporation) facilitates the intracellular delivery of administered drugs. For example, the cytotoxic effect of bleomycin is increased by more than 8000-fold [10].

Electroporation of the cell membrane causes significant vascular changes in the tumor region, inducing arteriolar vasoconstriction with the reduction of tumor tissue blood perfusion (vascular lock). This effect becomes irreversible when electric pulses are associated with chemotherapy (vascular disrupting effect), leading to an additional cascade. Tumor cell death, due to the effect of bleomycin, is a result of long-term lack of oxygen and nutrients and the accumulation of catalytic products. These effects also induce short-term cessation of bleeding and the palliation of hemorrhaging and ulcerated cutaneous lesions [11,12].

Currently, ECT indications include the local control of single and in-transit metastatic melanoma skin nodules not amenable to surgery or isolated limb perfusion or infusion, non-melanoma skin cancer, local recurrences and skin metastases from breast cancer, skin metastases from head and neck cancer and local recurrences in the oral cavity [13-19]. Since the first clinical experience of Mir et al. (1991) [20], a number of studies have shown that ECT is associated with a good overall response rate (92-99%), with a complete response rate of between 53% and 89%, without serious negative adverse events [6,7,21,22]. Quaglino et al. (2008) reported an overall response rate in 93% of patients with cutaneous metastases and improved local tumor control with repeated ECT. Moreover, in metastases >1 cm [2] which had lower response rates after the first ECT, a good antitumor effect was observed after retreatment with a complete response rate for 87% of the treated lesions after multiple ECT sessions [6]. Another advantage of ECT is the possibility to treat wider areas than the targeted metastatic lesion region, in order to avoid locoregional relapse. In addition, ECT of the lymphatic network surrounding the single metastatic nodule can improve local tumor control as demonstrated in patients with long-lasting response [23].

In the local control of cutaneous and subcutaneous metastatic lesions, ECT overcomes the low efficacy of classical chemotherapy and may mean surgery can be avoided. However, to our knowledge, its use has never previously been described in a neoadjuvant fashion to reduce the surgical extension in patients with metastatic melanoma.

Interestingly, ECT has been employed as a tissue-sparing treatment to reduce tumor burden for further treatments and surgery in other tumor types. One report described the use of preoperative ECT of anal melanoma to enable surgical resection with organ and function-sparing effect while a second case described ECT of a digital chondrosarcoma to avoid finger amputation [24,25]. In both cases, ECT appeared to be an effective neoadjuvant treatment for maintaining organ function and reducing the extent of surgical intervention. The favorable outcome of using neoadjuvant ECT for a metastatic melanoma nodule in the case presented here was evident as early as three weeks after the first session, with an 18-FDG PET-CT scan revealing a rapid four-fold decline in biological tumor activity from the baseline assessment as measured by SUVmax. Conservative surgery was carried out three months after the second ECT session and, to date, the patient remains disease-free with good functional and aesthetic outcomes (Figure 3). ECT was safely repeated on the basis of the positive response obtained after the first session. Moreover, as a consequence of the bleomycin mitotic cell death process, ECT was effectively targeted towards cancer cells, sparing adjacent healthy tissue of the cheek. ECT can therefore be applied to improve the patient’s quality of life, independent of life expectancy, to heal painful or bleeding lesions and also to preserve patients’ appearance and social interactions. A systematic review by Kis et al. (2011) similarly concluded that ECT in cutaneous melanoma is easy to perform without the potentially undesirable side effects of systemic chemotherapy and does not cause significant organ dysfunction or permanent disfigurement [19].

## Conclusions

ECT represents a safe and effective therapeutic approach that is associated with clear benefits in terms of quality of life (minimal discomfort, mild post-treatment pain and short duration of hospital stay) and may, in the neoadjuvant setting as reported here, offer the option of more conservative surgery and an improved cosmetic effect with complete local tumor control.

## Consent

Written informed consent was obtained from patient for publication of her clinical details and accompanying images. A copy of the written consent is available for review by the Editor-in-Chief of this journal.

## Competing interests

The authors declare that they have no competing interests.

## Authors’ contributions

NM enrolled the patient and drafted the manuscript. CC1 collected data and followed the patient’s course. SM performed surgical procedure. GDM partecipated surgical procedure. GB performed pathology studies. PAA performed electrochemotherapy. CC4 performed imaging studies. LA performed imaging studies. All authors read and approved the final manuscript.
